# Social responsibility in research and innovation practice and policy across global regions, institutional types, and fields: Interview data and qualitative content analysis outputs revealing the perspectives and experiences of professionals

**DOI:** 10.12688/openreseurope.15688.1

**Published:** 2023-04-28

**Authors:** Chris Foulds, Rihab Khalid, Eric A. Jensen, Obehi Sule, Lars Lorenz

**Affiliations:** 1 Global Sustainability Institute, Anglia Ruskin University, Cambridge, UK; 2 Lucy Cavendish College, University of Cambridge, Cambridge, UK; 3 Institute for Methods Innovation, Dublin, Ireland; 4 Thriving Natural Capital Challenge Centre, Scotland’s Rural College (SRUC), Edinburgh, UK; 5 School of Management, Anglia Ruskin University, Cambridge, UK; 6 Policy Research Unit, International Consortium of Research Staff Associations, Cork, Ireland

**Keywords:** RRI, Socially responsible science, Societal challenges, Researchers, Scientists, Global South, Research systems

## Abstract

The European Commission-funded RRING (Responsible Research and Innovation Networked Globally) Horizon 2020 project aimed to deliver activities that promoted a global understanding of Socially Responsible Research and Innovation (RRI). A necessary first step in this process was to understand how researchers (working across Global North and Global South contexts) implicitly understand and operationalise ideas relating to social responsibility within their day-to-day work. Here, we describe an empirical dataset that was gathered as part of the RRING project to investigate this topic. This Data Note explains the design and implementation of 113 structured qualitative interviews with a geographically diverse set of researchers (across 17 countries) focusing on their perspectives and experiences. Sample selection was aimed at maximising diversity. As well as spanning all five UNESCO world regions, these interview participants were drawn from a range of research fields (including energy; waste management; ICT/digital; bioeconomy) and institutional contexts (including research performing organisations; research funding organisations; industry and business; civil society organisations; policy bodies). This Data Note also indicates how and why a qualitative content analysis was implemented with this interview dataset, resulting in category counts available with the anonymised interview transcripts for public access.

## Plain language summary

Research and innovation are commonly advocated by policymakers and other groups as being central to societal prosperity, not least in addressing major challenges such as climate change. As such, there continues to be significant investment globally into research and innovation, with an increasing focus on providing new solutions for societal problems. However, to what extent are research and innovation professionals ensuring that their work is ‘responsibly’ delivered? And what does ‘responsibility’ even mean? And how may such understandings and definitions differ across the world?

In exploring these issues, we undertook 113 interviews with research and innovation professionals working across 17 countries. We asked them to tell us about their day-to-day work: what they practically did; who they worked with; what they saw success as looking like; how their work connected to society; challenges from government and their own organisations; procedures that they needed to adhere to; *etc*. From these discussions, we gained insights into a range of professional perspectives and experiences, including what they may mean for more ‘responsible research and innovation’. This Data Note presents how we designed, developed, undertook and analysed the interviews.

## Introduction

The European Commission-funded RRING (Responsible Research and Innovation Networked Globally) Horizon 2020 project aimed to deliver activities that promote a global understanding of responsible research and innovation (RRI), including launching a global network around such ideas. However, a necessary first step was to understand how research and innovation professionals (working across Global North and Global South contexts) implicitly understand and operationalise ideas of responsibility within their day-to-day work.

RRING therefore undertook structured qualitative interviews to gain a ‘bottom-up’ perspective on RRI, revealing existing local practices and policies that underpin research and innovation globally, in addition to how such practices and policies may need to change to better align with societal needs and values. Fundamentally, this required insights on perspectives, processes and practices from professionals employed around the world, to ensure that the organisation of research and innovation is ethical, socially inclusive and suitably addresses public concerns. The qualitative data presented here was analysed and reported alongside a large quantitative survey dataset in a major RRING project deliverable ( Jensen *et al.*, 2021). This empirical research is connected with the project’s policy agenda, which leveraged and bolstered UNESCO’s global policy instrument on RRI, called the Recommendation on Science and Scientific Researchers ( Jensen, 2022a; Jensen, 2022b).

Our intention in publishing this Data Note is two-fold. Firstly, the move to considering RRI beyond its Eurocentric roots is in its infancy, and thus we are expecting a potential ballooning of research in this area. We hope that our data, resources and the detailed procedures outlined in this Note are therefore of future (re)use. Secondly, given our interest in research responsibility, it is only appropriate that we ourselves follow high standards in transparency and open access.

## Methods

### Research instrument: structured interviews

Structured interviews were selected as the most consistent method for collecting additional in-depth data on RRI practices across the 17 countries. Consistency in the lines of questioning (including allowable follow-up questions) across the countries was considered particularly important given the range of interviewer experience. Each interview involved nine sets of questions, and specific interview protocol guidelines were provided to interviewers on how the interview was to be conducted.

Interviews were conducted either face-to-face or through virtual calls. Although face-to-face interviews allow for more personal contact and clarity in communication, virtual interviews were allowed where physical/financial limitations prevented face-to-face communication. The structured interviews generated reliable, focused, and uniform data relevant to producing a more comprehensive overview of current RRI practices globally.

### Research instrument design

***Country selection.*** To meaningfully attain an in-depth understanding of the ground-level experiences of research and innovation – from those who are actually performing those research and innovation roles – it was necessary to focus our efforts on specific countries in the world. The purpose of doing this was not to provide a representative sample of the world (or indeed its constituent regions), from which we could draw context-free conclusions of how (responsible) research and innovation is, or should be, done. Instead, the purpose was to tease out and qualitatively illustrate the range of research and innovation experiences across cultural contexts. This sub-section herein details our approach to country selection, through which this range was investigated.

The boundaries of country selection were steered by the search for sufficient spread across global regions, namely *via* the UNESCO world regions classification ( UNESCO, 2022): Europe and North America; Sub-Saharan Africa; Asia; Latin America and the Caribbean; and the Arab world. Due to the heterogeneity of the regions and countries around the world – and the many different aspects that RRI concepts involve, added to the local accessibility to data and local partners – the country selection was done on a multi-based criterion. Hence, in each region, the selection was based on the following five stages in the selection process:

Stage 1. Application of objective criteria to make initial selection:

- All countries were evaluated on the basis of their Gross Domestic Product (GDP) per capita in USD ( World Bank, 2019) and Gross Expenditure on Research and Development (GERD) ( UNESCO, 2019) to maximise sample diversity. One high and one low ranked country was selected for both GDP and GERD, with an alternative high/low country also identified for each GDP/GERD variable. The reasoning was to ensure that a range existed across the region in terms of domestic spend on research and innovation.
- A minimum population size of two million was set for country selection, as a proxy for ensuring that the respective country was large enough to have its own defined sector(s) conducting research and innovation.
- Only countries with a Travel Advisory Level of 1 and 2 were selected (except for Turkey), as per US State Department Travel advice. The rationale here was to ensure that our interviewers were safe and that also the political situation was stable enough in the respective country, so as to be able to draw conclusions on the support structures that are in place for research and innovation.
- The main exception is countries that were specified to be in the sample in the Grant Agreement ( *e.g.* US, India). These were indicated as ‘must select’ countries and further contributed to establishing a diverse sample.

Stage 2. Capacity of partners to collect data in initially selected countries

- Partners were canvassed to identify which selected countries they would be capable of helping with and/or lead on. In case no partner was available in the primary selected country, partner availability was determined for the alternative country, and a decision made accordingly.

Stage 3. Subcontracting alternatives for countries that project partners could not cover

- In countries where partners were not available to conduct interviews for both the primary selected and the alternative country, then University College Cork (as coordinator) and Anglia Ruskin University (as data collection lead) investigated options for a subcontract, with active input sought from partners and their networks.

Stage 4. Revisiting options if specific countries were too difficult to access

- If neither Stage 2 or Stage 3 were successful, then the next country on the list for the respective region and GERD/GDP variable was selected, with Stages 2 and 3 repeated. This was continued as needed until coverage was established.

Stage 5. Implementing contingencies should preferred solutions fail

- If a partner or subcontractor solution fell through during the setup, planning or early data collection phases ( *e.g.* negotiations on the subcontract itself failed), and a replacement could not be established, then Stage 4 was implemented again.

Based on this selection process, we selected four countries for each of the five world regions: one high and one low country for both GDP and GERD, for each region. These were locked in and pursued in earnest. However, a small number of these countries could not be included in the final dataset either due to unforeseen difficulties in undertaking the interviews ( *e.g.* one country’s central government would not formally allow the interviews to happen), or because of the data submitted did not meet the project’s quality thresholds required for analysis. Table 1 thus details the very final list of countries (including their GDP and GERD information) that were included in the final interview dataset.

**Table 1.** RRING focus countries included in the final interview dataset (n=113), based on regional stratification and a diverse economic and research system context in terms of GDP and GERD.

Regional distribution	Focus Country	Reason for selection
Europe and North America	United Kingdom	High GDP
	Italy	Low GDP
	USA	High GERD
	Israel	High GERD
	Serbia	Low GERD
Latin America and the Caribbean	Uruguay	High GDP
	Bolivia	Low GDP
	Brazil	High GERD
Asia	Singapore	High GDP
	India	Medium GDP
	Japan	High GERD
Arab World	Egypt	Low GDP
	Morocco	Low GDP
	Jordan	Low GERD
Sub-Saharan Africa	Botswana	High GDP
	Malawi	Low GDP
	South Africa	High GERD

***Participant sampling.*** The selection of participants from each country was based on standardised selection criteria, which each country’s interviewer team used as targets for participant recruitment:

- *Number of interviews:* A minimum of five interviews conducted per country.
- *Gender:* A 50-50 target split between men on the one hand and women and/or other gender identities on the other, with an acceptable minimum of 40% representation of women and/or other gender identities, per country.
- *Research fields:* At least one participant from each field category (ICT/digital; energy; waste management; bioeconomy) included in each country sample.
- *Institutional types:* At least one of each institutional type (research performing organisation; research funding organisation; industry and business; civil society organisation; policy body) included in each country sample.
- *Relevance of their professional work to the RRING project’s RRI interests:* Participants’ profiles/biographies/backgrounds needed to demonstrate experience of RRI-like activities, to ensure their professional work directly utilised (or at least complemented) research and innovation approaches.

This led to 113 RRI practitioners being interviewed, ranging from between the target minimum of five to a maximum of 11 participants per country, excluding Botswana and Singapore which only had three and two participants respectively. These interviews were undertaken over March to August 2019. Across the overall sample, interviewees comprised 43% women and 57% men. Eight countries (Uruguay, Bolivia, Brazil, Egypt, Morocco, Jordan, India, UK) had a minimum of one participant per institutional type, and, apart from USA and Singapore, there was a minimum of one participant across all four research fields per country. An anonymised participant breakdown is detailed in Table 2. 

**Table 2.** RRING interview participant breakdown, including information on research and innovation field, institutional type, gender and interview duration.

Country	Interview code	Interview duration (hrs:mins: secs)	Research & innovation field				Institutional type					Gender	
			*Energy*	*Waste* *Management*	*Information &* *Communications* *Technology*	*Bioeconomy*	*Research* *Performing* *Organisation*	*Research* *Funding* *Organisation*	*Industry* *&* *Business*	*Civil Society* *Organisation*	*Policy* *body*	*Man*	*Woman*
**UK**	GB01	00:58:50				✓			✓			✓	
	GB02	00:58:57				✓	✓						✓
	GB03	00:51:28			✓				✓				✓
	GB04	00:35:57	✓					✓			✓	✓	
	GB05	01:17:06				✓	✓					✓	
	GB06	00:49:27				✓	✓						✓
	GB07	01:15:27	✓	✓			✓					✓	
	GB08	00:44:33	✓							✓		✓	
**Italy**	I01	00:24:27			✓						✓	✓	
	I02	00:48:34	✓		✓				✓			✓	
	I03	00:58:40	✓		✓				✓			✓	
	I04	00:55:26			✓	✓			✓				✓
	I05	01:09:20		✓			✓				✓	✓	
**USA**	USA01	00:32:56	✓				✓					✓	
	USA02	00:48:57				✓	✓						✓
	USA03	00:17:51	✓				✓					✓	
	USA04	00:50:17				✓	✓						✓
	USA05	00:26:52				✓		✓				✓	
**Serbia**	SRB01	00:22:12	✓				✓					✓	
	SRB02	00:29:19			✓					✓			✓
	SRB03	00:31:58			✓					✓		✓	
	SRB04	00:34:39				✓			✓				✓
	SRB05	00:17:53		✓		✓	✓						✓
	SRB06	00:37:15				✓	✓					✓	
**Israel**	IL01	00:51:30		✓			✓					✓	
	IL02	00:56:41			✓		✓						✓
	IL03	00:51:42	✓	✓		✓	✓						✓
	IL04	01:03:56	✓		✓		✓					✓	
	IL05	00:43:13	✓	✓			✓						✓
**Botswana**	BW01	00:34:01			✓		✓					✓	
	BW02	00:23:49	✓	✓	✓	✓	✓	✓				✓	
	BW03	00:40:39	✓	✓	✓		✓					✓	
**Malawi**	MW01	00:46:36			✓	✓	✓			✓		✓	
	MW02	00:41:55			✓				✓			✓	
	MW03	00:27:32	✓				✓		✓			✓	
	MW04	00:18:56			✓		✓						✓
	MW05	00:22:27	✓	✓	✓				✓	✓			✓
	MW06	00:39:37				✓	✓					✓	
	MW07	00:32:21			✓		✓	✓					✓
	MW09	00:38:46			✓		✓					✓	
**South Africa**	ZA01	00:34:18	✓				✓		✓			✓	
	ZA02	00:35:58		✓		✓		✓			✓		✓
	ZA03	01:45:05	✓				✓						✓
	ZA04	00:15:10				✓	✓						✓
	ZA05	00:44:40	✓				✓				✓	✓	
	ZA06	00:18:24			✓		✓					✓	
	ZA07	00:18:24	✓	✓		✓	✓	✓			✓	✓	
	ZA08	00:30:12		✓			✓	✓					✓
	ZA09	00:38:21	✓				✓					✓	
	ZA10	01:14:02	✓				✓						✓
**India**	IND01	00:42:20				✓	✓					✓	
	IND02	00:46:37	✓		✓						✓	✓	
	IND03	00:47:00		✓	✓		✓			✓			✓
	IND04	00:41:00		✓			✓			✓			✓
	IND05	01:03:00	✓					✓		✓		✓	
	IND06	00:33:00	✓	✓					✓			✓	
**Singapore**	SGP01	01:12:38	✓				✓					✓	
	SGP02	01:08:47			✓		✓				✓		✓
**Japan**	J01	01:09:49			✓✓✓		✓✓	✓✓				✓	✓✓
	J02	01:27:44				✓	✓						✓
	J03	01:22:18			✓		✓						✓
	J04	01:09:49			✓		✓					✓	
	J05	01:05:58	✓	✓	✓	✓	✓						✓
**Uruguay**	ROU01	01:01:36				✓				✓		✓	
	ROU02	01:12:21	✓	✓		✓					✓	✓	
	ROU03	01:07:39			✓					✓		✓	✓
	ROU04	00:52:27	✓	✓		✓					✓		✓
	ROU05	00:36:51	✓	✓	✓	✓		✓					✓
**Bolivia**	BO01	00:42:37	✓				✓				✓	✓	
	BO02	00:56:09			✓		✓						✓
	BO03	01:12:43		✓			✓			✓		✓	
	BO04	00:52:21	✓				✓					✓	
	BO05	00:41:36				✓	✓			✓			✓
	BO06	01:26:17			✓				✓	✓			✓
	BO07	01:06:56				✓	✓						✓
	BO08	00:43:18		✓					✓			✓	
	BO09	01:19:13			✓		✓					✓	
**Brazil**	BR01	01:16:00				✓			✓				✓
	BR02	00:47:39	✓		✓		✓					✓	
	BR03	01:04:38	✓✓	✓		✓				✓✓		✓	✓
	BR04	01:32:30				✓	✓						✓
	BR05	00:54:56	✓	✓		✓				✓		✓	
	BR06	01:07:54				✓	✓	✓			✓	✓	
	BR07	00:54:56			✓	✓				✓		✓	
**Egypt**	EG01	00:56:01				✓	✓					✓	
	EG02	00:40:07	✓	✓	✓	✓		✓				✓	
	EG03	00:33:53			✓						✓		✓
	EG04	00:51:33	✓			✓	✓		✓	✓			✓
	EG05	00:22:23			✓		✓					✓	
	EG06	00:40:07	✓				✓						✓
	EG07	00:33:00	✓	✓	✓	✓		✓			✓		✓
	EG08	00:49:21	✓				✓					✓	
	EG09	00:58:32				✓	✓			✓		✓	
	EG10	00:40:33		✓					✓			✓	
**Morocco**	MO01	00:45:40	✓						✓			✓	
	MO02	00:20:33	✓		✓				✓	✓		✓	
	MO03	00:32:47			✓		✓					✓	
	MO04	00:16:46		✓			✓						✓
	MO05	00:30:35	✓				✓						✓
	MO06	00:27:33	✓	✓			✓			✓			✓
	MO07	00:39:12		✓		✓			✓			✓	
	MO08	00:56:58		✓					✓			✓	
	MO10	00:10:31			✓						✓	✓	
	MO11	00:31:21	✓	✓			✓					✓	
	MO12	00:35:24		✓			✓						✓
**Jordan**	HKJ01	00:38:06				✓	✓			✓			✓
	HKJ02	00:58:21	✓				✓						✓
	HKJ03	00:30:52			✓		✓	✓			✓		✓
	HKJ04	00:47:07		✓✓						✓✓			✓✓
	HKJ05	00:41:01	✓	✓	✓	✓	✓					✓	
	HKJ06	00:50:55		✓			✓					✓	
	HKJ07	00:58:21	✓				✓					✓	
	HKJ08	00:54:03	✓		✓		✓			✓		✓	
**Total**		**88:58:49**	**49**	**35**	**45**	**42**	**74**	**15**	**20**	**25**	**15**	**67**	**51**

***Protocol design and requirements.*** The priority for the interviews was to probe participants’ personal interpretations, perceptions and understandings of RRI-like practices, as part of their day-to-day professional work. In particular, the interview protocol’s questions focused on generating insights on the participants’ in-situ experiences and indeed their social construction of such RRI-like practices. In this way, the interview protocol was not designed to specifically target ‘factual’ evidence and information.

The interview protocol included nine sets of questions, organised across: types of research and innovation activities performed; public engagement; aligning with ethical values; open access and open data; meeting societal needs; anticipation; diversity and gender equality; responsibility; and, closing reflections. We also insisted that all interviewers did not mention “Responsible Research and Innovation”, “RRI” and even “responsibility” in the framing of the interview and/or in the phrasing of most questions, as we wanted to ensure participants maintained their focus on their own experiences, as opposed to *e.g.* being distracted by performative ideas of what they thought interviewers believed responsible practice entailed.

The interview protocol was peer-reviewed by three RRING colleagues who were not involved in the interview planning, implementation or analysis. The review focused on academic standards ( *e.g.* rigour, consistency, novelty). The protocol was additionally peer-reviewed by RRING’s Gender Sub-Committee, to ensure intersectional issues were adequately accounted for, both in terms of question content and interviewer guidance. This protocol is available in Foulds and Sule (2019).

Alongside the protocol, a fieldnotes form was circulated to all interviews (acting as interview memos). This short interviewer survey asked for brief reflections on participant familiarity with the terms used; the atmosphere during the interview; moments where the interviewer particularly influenced participant responses; reflections on the method used, in particular the structured nature of the interaction; *etc.etc.* These fieldnotes were completed as soon as possible after the interview by the interviewer(s) themselves. The fieldnotes had the dual purpose of being actual data, as well as providing context for the data analysis (all analysts were instructed to read the respective fieldnotes entry before coding each interview). The fieldnotes form template is available in Foulds and Sule (2019).

The interview protocol and fieldnotes template were piloted twice, in English. The two pilot interviews were conducted in the UK and in South Africa, both of which also formed part of the final dataset too. These pilots led to improvements to the protocol and interviewer guidance relating to, for example: question phrasing; precisely when and how one could deviate from the structured lines of questioning; and transcription requirements.

All interviews were audio recorded, transcribed, and then translated from local languages into English. Transcripts were written up, alongside the fieldnotes, as soon as possible post-interview and similarly submitted for quality assurance and consistency checks centrally as soon as possible too.

Anonymised versions of the 29 interview transcripts, for which permission was granted for public sharing, is available in RRING Project (2021).

### Data analysis and validation procedures

***Analysis approach: qualitative content analysis.*** Qualitative content analysis was used as the primary data analysis method. It focused on forming thematic categories through a consistent set of codes applied to textual data (in this case, transcripts and fieldnotes) ( Morgan, 1993; also see Jensen & Laurie, 2016). Content was analysed both descriptively and interpretatively, with the spotlight put on the thematic categories and codes with the highest prevalence across all the interviews. The analysis was led by the second author of this paper.

The coding and analysis of the interviews took place across five phases, which are now detailed in turn. Analysis of the qualitative data was done using NVivo 12.

Analysis Phase 1: Inductive coding and preparation of the codebook.

Inductive coding was conducted using a grounded theory approach. Following guidelines in Bazeley and Jackson (2013), Miles *et al.* (2014) and Saldana (2016), various stages of coding and recoding were done for progressive refinement of the codes generated, divided into two cycles. In the first cycle of coding, an eclectic combination of attribute, structural, descriptive, in vivo, value, versus and holistic/lumper codes, were used. Coding was led by the objective of identifying best practices in research and innovation and determining the participants’ perspectives on the various structured interview themes. In the second cycle of coding, thematic codes were used to categorise the sub-level codes into higher-level themes identified within the context of the research objective. In this stage, although the interview structure guided the formation of themes, it was not used deductively to generate categories. As a result, other cross-cutting themes like ‘Conflicts in theory and practice’ and ‘Collaboration’ also emerged.

Initially, pilot coding was carried out for two interviews. Based on this analysis, a pilot version of the codebook was prepared. This was then peer-reviewed and subsequent revisions were made. After this, a preliminary codebook was prepared based on the qualitative analysis of 30 interviews. This codebook contained 257 codes under 13 categories. The coding was done to account for both cross-cutting (i.e. across all the interview questions and all the geographies/fields/ *etc.etc.*) themes ( *e.g.* enablers, constraints, conflicts, *etc.etc.*), as well as context- and question section-specific subject matter based on the structured interview-based themes ( *e.g.* public engagement, open science, *etc.etc.*). After subsequent peer reviews (by first and third author of this paper), revisions were made to the codebook, including tackling boundary issues, complexity, sheer number of codes, coding instructions, *etc.etc.* This revised version, which contained 117 codes under 12 categories, was then used in the coder training phase.

The codebook’s 30 interviews were selected from 11 countries to ensure a good distribution of country representation and, within each country, at least one interview from each gender was selected. Of the 30 interviews analysed, approximately 40% of interviews were with women. In addition, all research and innovation fields and institutional types were covered in a fairly even distribution. An anonymised version of the final codebook is available in Foulds *et al.* (2019).

Analysis Phase 2: Coder training.

Coding of the remaining 84 transcripts was done deductively by a team of three coders (in addition to the Lead Coder, also this paper’s second author), using the codebook from Phase 1. For this, the coders were provided with extensive training in two practice rounds. In the first round, a full-day training workshop was held that included the methodological lead (paper first author), all four coders, and an observer from one of the partner organisations responsible for coding quality assurance. The coders were given sufficient time to go through the codebook and familiarise themselves with all the codes in advance. In the first part of the workshop, the codebook and the coding process were further explained to all the coders, giving them the opportunity to discuss and ask questions wherever necessary. In the second part, the coders were given a pre-prepared practice transcript with coded text highlighted and bracketed in different colours with blank spaces for inserting codes. This was done in accordance with the method proposed by Campbell *et al.* (2013) to determine inter-coder agreement.

In the last part of the workshop, the coders submitted their coded transcripts, which were then compared to determine inter-coder agreements. The coders discussed their common experiences and compared notes to better understand the codes and how to use the codebook deductively moving forward. Based on these discussions, further improvements were made to the codebook, relating to guidance on *e.g.,* simultaneous coding, length of coding, repetition of text, making inferences, boundaries between codes, and coding gaps.

In the second practice round, each of the four coders was given a separate second practice transcript to be coded independently. Coding was then compared with the Lead Coder over virtual calibration meetings, and inter-coder agreement determined and reached. It was found that percentage agreement with one coder was below the minimum standard of 61%. Additional training was therefore carried out with that one coder (Coder 3). A new practice transcript was provided to Coder 3, and inter-coder agreement was again determined with the Lead Coder. The two coders then discussed their coding through another virtual calibration meeting, reaching an agreement of 82%. This second practice round also led to some minimal revisions to the codebook, mainly concerning the ambiguity of certain definitions.

Analysis Phase 3: Deductive coding.

The finalised codebook from Phase 2 was used by the three additional coders to deductively code the remaining 84 interview transcripts. An NVivo shell file was provided to ensure consistency in the deployment of the coding scheme. Regular review and feedback sessions were also conducted periodically during the deductive coding phase, between each individual coder and the Lead Coder.

While coding for the remaining 84 interviews was mainly to be done deductively, the coders were expected to flag any critical new codes and reach a satisfactory inter-coder agreement. The distribution of interview transcripts was done through a just and fair process. Initially, 10 interview transcripts were allocated to each coder, distributed numerically based on interview code. After the initial distribution, subsequent transcript allocations were based on a first-come-first-served basis: coders who completed their coding task faster were consequently allotted a higher number of interviews.

Analysis Phase 4: Inter-coder reliability checks.

The final statistical assessment of inter-coder reliability was conducted on about 21% of interviews (18 interviews) using Krippendorff's Alpha (also called Krippendorff's Coefficient) ( Krippendorff, 2011). The values for this intercoder reliability analysis were calculated using Krippendorff’s Alpha Python implementation ‘fast-krippendorff’ ( Pln-Fing-Udelar, 2019, no pagination). Since values were given as the frequency of a variable’s occurrence, the interval metric for Krippendorff’s Alpha was used. This accounts for the interval scale that is being used, meaning that the difference between one and 10 is weighted more severely than the difference between nine and 10 in the application of the statistical test.

Initially, nine interviews (about 11% of interviews coded) were selected from the 84 deductively coded interviews for inter-coder reliability testing. These nine interviews were chosen through random sampling, ensuring a proportional distribution of interviews from each coder based on the total number of interviews coded. Table 3 presents an overview of this distribution and selection process. Excel’s random number generator was used to randomly generate the number of the interview to be tested. The results of the inter-coder reliability test for each code are presented in Foulds *et al.* (2019).

**Table 3.** Transcript distribution and selection of interviews for inter-coder reliability testing.

**Coder Number**	Coder 2	Coder 3	Coder 4
**No. of interviews coded**	30	10	54
**No. of interviews tested (20% of interviews coded)**	6	2	10
**Randomly generated interview (number) code for** ** testing inter-coder reliability**	SGP02; BO01; ROU03; USA02; SRB01; BO06	EG05; GB05	BW01; I02; MW03; USA04; MO04; IL05; MO02; MO03; MO12; ZA05

The initial inter-coder reliability analysis found that only four of 117 codes had a Krippendorff’s Alpha value below the commonly accepted threshold of 0.8. These were “71: Personal responsibility and morality” (0.79), “88: Anticipation” (0.77), “91: Responsive approach” (0.78) and “117: Difficulties in collaboration and engagement” (0.74). For 15 codes, an alpha score could not be successfully calculated, as these were not used during coding, and a code count greater than 0 is required to calculate Krippendorff’s Alpha. Arguably, this also represents a perfect agreement, as both coders decided not to code the variable ever. However, it is usually advisable to either extend the sample size for the inter-coder reliability analysis to increase the probability of encompassing these codes, or to decide not to use the variable for further analysis, should it be detected, as the reliability test does not evidence the coders’ ability to independently detect its presence, but only its absence. Hence, another nine interviews were randomly selected from the 84 interviews, to get a total test sample of about 21%. The inter-coder reliability analysis was then repeated using a test sample of 18 interviews.

This final test showed that only seven of 117 variables yielded an alpha value below the commonly accepted reliability threshold of 0.8. These were “40: Empowerment tools” (0.78), “42: Campaigning-Lobbying” (0.75), “53: Diversity and inclusion” (0.76), “56: Gender diversity” (0.72), “57: Ethnic and religious diversity” (0.77) and “67: Discrimination- a non-issue” (0.71). We still proceeded with these seven codes, as they were in the zone of acceptability.

Additionally, the code “113: Ecosystem of support” (0.41) yielded an unacceptably low alpha value, leading to its rejection for further analysis due to its poor reliability. As this is the only code with a drastically lower alpha value, it can be considered as an outlier and does not impede the reliability of other codes. This is the only code that we excluded from further analysis.

For six codes ^1^, an alpha score could not be successfully calculated, as a code count greater than 0 was required to calculate Krippendorff’s Alpha, meaning the variable was never present in the coded data, according to both coders. Given that the recommended sample proportion for inter-coder reliability had already been exceeded with an inclusion of about 21% of all data, it was deemed reasonable to proceed with these categories which are simply relatively low prevalence in the sample. Therefore, these six codes were retained for full coding and analysis.

On average, coders achieved a Krippendorff’s Alpha value of 0.95, and a reliability of over 0.8 for 89% of variables. These are good results overall, indicating a robust coding process.

Analysis Phase 5: Code counting within themes.

We categorised the codes into themes, inspired by: the six RRI pillars (public engagement, open access and data, science education, ethics, gender equality, and governance), as defined by the RRING project and European Union's Framework Programmes under the European Commission; and Lubberink *et al.*’s (2017) AIRR dimensions (anticipation; inclusion; reflexion; responsiveness) that look at the intertwining of related concepts of RRI, social innovation and sustainable innovation. This led to the codes being clustered into seven themes to cover a wide range of aspects of socially responsible research and innovation: gender equality and inclusivity; public engagement; open science; anticipative, reflective and responsive RRI; science education; ethics; and governance of RRI.

In each of these seven themes, code counting was done at the level of UNESCO global regions. Code counts for these major themes provided the first step for analysis; an efficient review and comparison of code counts highlighted the most prevalent themes during the interview conversations, while also pointing to issues which (if at all) had been undermined or paid less attention. This helped in targeting key codes for further qualitative interrogation and interpretation. For this, four codes with the greatest prevalence across all 133 interviews (as indicated by having the highest counts) were selected for a more in-depth analysis of each theme. All code counting results are presented in Foulds *et al.* (2019).

Within these codes that were identified based on their prevalence, the associated code interview text was then interrogated and analysed more deeply. Preliminary findings are included in the RRING project deliverable report that this work fed into ( Jensen *et al.*, 2021).

## Ethics policies and informed consent

The data collection and analysis methods were approved by the Departmental Research Ethics Panel located within Anglia Ruskin University’s Global Sustainability Institute (reference number of GSIDREP-1819-003; approval date of 28 November 2018). This process ensured ethics experts signed off on, for example, the Participant Information Sheets and Informed Consent forms.

Informed consent was obtained for all participants, prior to their data being included in the analysis and any subsequent publication, as per the EU General Data Protection Regulation requirements. Consent was obtained pre-interview through an email correspondence, where the participant was asked to print, sign and scan the consent form, and email it back to the interviewer after reading the Participant Information Sheet. If this approach was not viable, the Participant Information Sheet was read before the interview and consent was instead audio recorded. Whilst all participants (n=113) consented to the data being anonymously used in analyses and in our final publications, only 26% (29 participants) consented to the anonymised transcripts being published in an open access data portal.

## Data Availability

The project contains the following underlying data ( https://doi.org/10.5281/zenodo.5070359): 29 anonymised interview transcripts, spanning eight countries. Specifically: Bolivia (seven transcripts); India (two transcripts); Japan (two transcripts); Malawi (four transcripts); United Kingdom (five transcripts); Serbia (five transcripts); Singapore (one transcript). The project contains the following extended data ( https://doi.org/10.5281/zenodo.7404599): Interview protocol guidelines. Fieldnotes (interview memo) template. The project contains the following interview analysis and validation outputs ( https://doi.org/10.5281/zenodo.7404643): Interview codebook, including theme/code/sub-code definitions and anonymised illustrative examples. Code counts covering all codes across all 113 interviews, including organisation at both themes and world regions. Inter-coder reliability data and test results. Data are available under the terms of the Creative Commons Attribution 4.0 International license (CC-BY 4.0).
