# Supplementary material for: Social responsibility in research and innovation practice and policy across global regions, institutional types, and fields: Interview data and qualitative content analysis outputs revealing the perspectives and experiences of professionals
Source: Open Res Eur. 2023 Apr 28;3:65. [Version 1] doi: 10.12688/openreseurope.15688.1 (PMC10445911; doi:10.12688/openreseurope.15688.1)
Supplement: Peer review history

# Peer review: Reviewer response for version 1

**DOI:** 10.21956/openreseurope.16949.r31446

**Published:** 2023-07-19

**Authors:** Poonam Pandey

**Affiliations:** 1 Post-Growth Innovation Lab, Universidade de Vigo, Vigo, Galicia, Spain

This data note on 'Social responsibility....experiences of professionals' is a methodologically sound description of the data collection and data analysis protocol that the authors followed. In my view such scholarly products can act as useful guides for researchers who are undertaking similar scholarly exercises. I recommend that the note should be indexed with minor revisions along following lines:

1. It would be very useful if the authors could add a separate section on the limitations and challenges of using a qualitative data collection approach that is too focused on harmonization and standardization. For example, it would be nice to reflect on the use of methodological tools such as ‘Krippendorf’s alfa value’ and what negotiations are made during making the data more ‘inter-coder reliable’? Is there a compromise, in terms of the depth and richness of interpretation, when one chooses to deal with qualitative data in large quantity. It would also be useful to reflect a little on the biases embedded in the software, and the ethical challenges implied when individual coders analyse the data of a context for which they do not have much understanding of its socio-economic, cultural, historical, and political settings.
2. The choice of countries based on GDP and GERD in each region is not symmetrical. Its not clear why some countries are chosen because they have high/low GDP and why some countries are chosen because they have high/low GERD. A lot goes between different permutations and combinations of GDP and GERD that needs to be explained by political, historical, and socio-cultural factors. A clear reasoning should follow the choice of these countries and their GERD and GDP.
3. I looked at the additional interview data for India (two interviews). It appears that the same interview is uploaded twice! I request the authors to double check any similar discrepancies.

Are sufficient details of methods and materials provided to allow replication by others?

Yes

Is the rationale for creating the dataset(s) clearly described?

Yes

Are the datasets clearly presented in a useable and accessible format?

Partly

Are the protocols appropriate and is the work technically sound?

Yes

Reviewer Expertise:

Responsible Research and Innovation; Science, Technology and Innovation Policy; Developmental politics; global south; STS

I confirm that I have read this submission and believe that I have an appropriate level of expertise to confirm that it is of an acceptable scientific standard, however I have significant reservations, as outlined above.

# Peer review: Reviewer response for version 1

**DOI:** 10.21956/openreseurope.16949.r31583

**Published:** 2023-06-29

**Authors:** Ivan Buljan

**Affiliations:** 1 Department of Psychology, Faculty of Humanities and Social Sciences, University of Split, Split, Croatia

Thank you for the opportunity to review this, in my opinion, very detailed paper. 

 I have very little to add to what authors already stated in their work. 

 The paper presents, in great detail, the procedures undertaken to collect, analyse and interpret the data related to responsible research and innovation. The authors provide publicly available materials from their work.

 My suggestions are more related to clarity of the text presented. 

- First, although being a data note, I would argue that including a reporting checklist (e.g. COREQ) would give readers more easier ways to navigate the text. It would be better to specify which sentences are referring to each element.
- Moreover, although it is not usual in qualitative research, I think that a flowchart of the process would help greatly in understanding of the analysis phases.
- Table 2 could be removed to the end of the text, or placed as Supplementary file.
- Also, instead of referring to the author order, maybe adding initials of researchers would be preferred, as well as description of their experience and background.
- Also, there are several typos in the text which could be corrected (repetitive etc. on p11).

Once again, thank you for opportunity to review this manuscript.

Are sufficient details of methods and materials provided to allow replication by others?

Yes

Is the rationale for creating the dataset(s) clearly described?

Yes

Are the datasets clearly presented in a useable and accessible format?

Yes

Are the protocols appropriate and is the work technically sound?

Yes

Reviewer Expertise:

Psychology; Research integrity: Research methodology

I confirm that I have read this submission and believe that I have an appropriate level of expertise to confirm that it is of an acceptable scientific standard.

# Peer review: Reviewer response for version 1

**DOI:** 10.21956/openreseurope.16949.r31585

**Published:** 2023-06-29

**Authors:** Håkon Finne

**Affiliations:** 1 Technology Management, SINTEF, Trondheim, Trøndelag, Norway

**Introduction**

 The data note provides metadata for 113 structured qualitative interviews collected across 17 countries around how Responsible Research and Innovation (RRI) is conceptualized and practiced. The data derive from the RRING project (rring.eu) to promote a global understanding of RRI.

 Interview transcripts - such as those shared in the data set described in this data note - are notoriously difficult to use productively outside the context and process of their original collection or construction. It is therefore interesting to see how the data note describes this context and process in great detail. At first this description reads as an attempt at purging the interviews and the subsequent analyses of any subjectivity on the part of the investigators. This would pave the road for future replications. It might also give the categories (codes) developed inductively a near context-free character, as implicitly required in most quantitative analyses. At the very least, however, I think it is fair to read this process as an attempt at securing that the variations in the data stem from the interviewees and not from the interviewers and the analysts, who themselves span a great variety of contexts and approaches. The data note, then, can be read as a description of both the methodology of constructing the data and of the data itself.

 For my review, I will take a slightly different approach. The data set merits a brief structural description, adapted to the perspective of this review. In my perspective, it has four layers of data, which we may call construction data, interview data (transcripts), codes (or categories), and code counts (or category counts).

**Construction data** corresponds to the description of procedures for generating the collection of interview data. These procedures are meticulously described in such a way that they are suitable for replication by other research teams. They also have a data character because they give a basis for deconstructing the protocols for sampling and interviews, should someone wish to really test the robustness of a study that aims to broaden the cultural basis for the very important (and very fluid) concept of RRI. For example, in order to study how the processual aspects of the EU commission's six RRI policy pillars are balanced against (or integrated with) RRI as a matter of goals and outcomes (which also occurs in the interviews). Or, in the words that the authors themselves use when describing the variation between interviewees, to understand how the authors " *implicitly understand and operationalise*" their own research in this project.

**Interview data** is usually considered the primary data of such a study and could also be called the data proper. The full data set contains transcripts of 113 structured qualitative interviews, 29 of which are made available in the publicly available data set.

 The **codes** are a form of analytically based thematization of the interview data, generated in this project through a grounded-theory approach, guided by the analytical purpose of the project (i.e., an investigation of the global variety of "best RRI practices" in actual R&I). The inductive source of the codes in the interviews themselves make them data in their own right. In line with requirements of a qualitative content analysis, 116 codes grouped under 12 headings are developed in this way and then applied to all 113 interviews as a way of data reduction. The detailed coding of sections of the text is not shown in the 29 transcripts.

**Counts** of the occurrence of these 116 codes are then generated across all 113 interviews and their contexts (i.e. gender, country (and UNESCO region), the four sampled R&I fields, and institutional type or role in R&I system). This makes the code counts an appropriately reduced dataset for a qualitative content analysis, i.e. in principle without having to consider the interview transcripts themselves. This data reduction is described in the data note and performed in a different paper. These counts are made available in a spreadsheet with a 113x116 count table and a number of tables that are aggregated over two or three levels across country (and region), R&I fields (renamed as themes), and institutional type (renamed as stakeholders) and also across the 12 groupings of codes.

**Is the rationale for creating the dataset(s) clearly described?**

 The rationale for collecting the full dataset in the project is clearly described: To empirically understand how researchers (and other relevant actors) in a wide array of contexts " *implicitly understand and operationalise ideas relating to social responsibility within their day-to-day work*".

 The rationale for making the subset available (the protocols, the 29 transcripts where the interviewee allowed anonymized publication, the 116 codes and their counts across the 113 interviews) is to enable future (re)use of the data, resources and detailed procedures for generating the data and the codes in a potentially ballooning field of research. Also, the authors wish to practice social responsibility in their own work by following high standards of transparency and open access.

**Are the protocols appropriate and is the work technically sound?**

 Absolutely, given the purpose of the project and its chosen strategies.

**Are sufficient details of methods and materials provided to allow replication by others?**

 Data generating methods (including procedures) are meticulous, and meticulously described. Because the multi-stage sampling is strategically honed for variation in significant contexts and not for creating a random sample, replications as grounds for a quantative meta-analysis should hardly be expected. Rather, the value of the procedural documentation is that it may make it easier not to lose the connection between the code counts and the actual interviews. In theory, this should open for alternative analyses despite the unavailability of the major part of the interview transcripts.

**Are the datasets clearly presented in a useable and accessible format?**

 The **construction data** are as clearly presented as any.

 The **interview transcripts** are useable and accessible. The only problem is the sample self-selection bias (willingness to release the transcripts), but 29 is definitely more useful than 0.

 The **codes** (the what) are meaningful and relevant for the project's purpose and for related research interests.

 The **code counts** (the what and how many) should be read as data for a qualitative content analysis in line with Morgan's (1993) recommendation, i.e. to be used as a basis for investigating why and how these counts appear in the data. Because the sample is not random, other uses should be carefully considered, as the data note also indicates (at least indirectly). This may limit its appropriate usability. To work with the aggregated tables rather than with the full 113x116 dataset is cumbersome. This is illustrated by the 1,200 pages long project report (Jensen et al., 2021), where hundreds of pages are dedicated to discussing said tables, and - significantly - enhanced by quotations from interviews not available in the public datasets. Many analysts would say that this demonstrates the difficulty of using the code counts alone as a reduced data representation of the 113 interviews. However, this would depend on the purpose of the analysis. Many analysts would simply prefer the full code count dataset (113x116 counts) to include the background variables to make it 113x129 (with binary variables for gender, R&I field, and stakeholder because some interviews had more than one interviewee and some interviewees represented multiple contexts). Then they would also be free to use the aggregation and statistics software of their choice to look for patterns in the data, including co-occurrence, which is not readily visible in the aggregated tables in the provided spreadsheet.

 Finally, I have some minor comments on the actual text of the data note, all intended to enhance **readability**.

- The UNESCO region that comprises Asia does also include the Pacific, but it is only named Asia in the text. The use of full official terms in the text will enhance clarity.
- Participant sampling, relevance: It is unclear whether " *experience of RRI-like activities*" excludes individuals who are mainly researching RRI as a phenomenon. This is clarified in the following section ("RRI practitioners"), but a slight rewording might improve the flow of the argument.
- A fieldnotes form was "circulated to all interviews" (p. 5). I expect this is a typo for "interviewers".
- Transcripts were " *submitted for quality assurance and consistency checks centrally*" (p. 11). I read this to be a check with the interviewees until the word "centrally" appeared. Moving "centrally" to between "submitted" and "for" would steer away from the alternative interpretation of interviewees reviewing what they said in their interviews.
- Code counts pointed to issues which " *had been undermined*" (p. 13). At first I wondered how interviews that were so carefully planned could have sabotaged the surfacing of important issues. Then I understood that it might be a question of digging less into those issues than the occasion would have permitted (sub-optimally mined). A different word might clarify the meaning.

**Conclusion**

 In conclusion, I believe the main re-use of the data (at all four levels) will be in interpreting the analytical and published output from the project and the generation of new ideas for related research. Which would be no mean feat. I would recommend indexing of the data note and a slight re-organization of the code count data to include the background variables in the dataset at interview level as described above (i.e., to be a 113x129 table).

**References**

 Jensen, Eric et al. (2021): State of the Art of RRI in the Five UNESCO World Regions. Zenodo. DOI: 10.5281/zenodo.4926175

 Morgan, David L (1993): "Qualitative Content Analysis: A Guide to Paths Not Taken". Qualitative Health Research. DOI: 10.1177/104973239300300107

Are sufficient details of methods and materials provided to allow replication by others?

Yes

Is the rationale for creating the dataset(s) clearly described?

Yes

Are the datasets clearly presented in a useable and accessible format?

Partly

Are the protocols appropriate and is the work technically sound?

Yes

Reviewer Expertise:

My relevant areas of research include studies of innovation processes and systems, the functions of research and innovation in society, policies and practices of sustainable industry development (including smart specialization), and worker participation in technological development. Relevant methodological approaches include quantitative and qualitative studies, participatory action research, and impact and process evaluation of public programs.

I confirm that I have read this submission and believe that I have an appropriate level of expertise to confirm that it is of an acceptable scientific standard.

## References

[1] : State of the Art of RRI in the Five UNESCO World Regions (1.0). *Zenodo* .2021; 10.5281/zenodo.4926175 10.5281/zenodo.4926175 Reference source

[2] : Qualitative content analysis: a guide to paths not taken. *Qual Health Res* .1993;3(1) : 10.1177/104973239300300107 112-21 10.1177/104973239300300107 8457790
